# Rapid evaporative ionization mass spectrometry in surgery: a systematic review

**DOI:** 10.1093/bjs/znaf228

**Published:** 2025-11-12

**Authors:** Angus R J Barber, Alexander Dottore, James Leigh, Mark Fear, Fiona Wood

**Affiliations:** Plastic Surgery Department, Sir Charles Gairdner Hospital, North Metropolitan Health Service, Perth, Western Australia, Australia; Curtin Medical School, Curtin University, Perth, Western Australia, Australia; School of Biomedical Sciences, University of Western Australia, Perth, Western Australia, Australia; Orthopaedic Surgery Department, Royal Adelaide Hospital, Adelaide, South Australia, Australia; Faculty of Medicine and Health, University of Sydney, Sydney, New South Wales, Australia; Nuffield Department of Primary Care Health Sciences, University of Oxford, Oxford, UK; Burn Injury Research Unit, School of Biomedical Sciences, University of Western Australia, Perth, Western Australia, Australia; State Adult Burn Unit, Fiona Stanley Hospital, Murdoch, Western Australia, Australia; Fiona Wood Foundation, Perth, Western Australia, Australia; Burn Injury Research Unit, School of Biomedical Sciences, University of Western Australia, Perth, Western Australia, Australia

## Abstract

**Background:**

Rapid evaporative ionization mass spectrometry (REIMS) is an emerging technology facilitating real-time intraoperative tissue identification during surgery. This review aims to discuss the applications and reported outcomes of REIMS technology in a surgical context.

**Methods:**

A systematic review was performed using four electronic databases that were searched in August 2025: MEDLINE, Emcare, Embase, and Web of Science. Eligible studies were peer-reviewed, included five or more patients, and evaluated REIMS technology in the context of a surgical specialty or pathology. Two independent reviewers screened studies, extracted data, and assessed risk of bias using the QUADAS-2 tool. The study protocol was registered in the PROSPERO international prospective register of systematic reviews before commencing the review (CRD42024546741).

**Results:**

A total of 344 records underwent initial screening, with 26 studies included. Included articles originated from seven countries and applied REIMS to eight surgical specialties. Twenty-three of the included articles used REIMS to identify cancerous tissue. All included studies reported both qualitative and quantitative outcomes. Included studies demonstrated a variety of surgical applications with promising results with regard to accuracy, sensitivity, and specificity. Both *ex vivo* and *in vivo* applications were explored, but limited *in vivo* data was reported and logistical limitations were identified.

**Conclusion:**

Most of the evidence supporting the use of REIMS in surgery originates from an *ex vivo* environment. Current limitations of the technique include equipment logistics and the complexity of interpretation of data and further *in vivo* studies with larger patient numbers are required to support more widespread application.

## Teaser

Rapid evaporative ionization mass spectrometry (REIMS) is an emerging technology facilitating real-time intraoperative tissue identification during surgery. This review discusses the applications and reported outcomes of REIMS technology in a surgical context, by analysing 26 included articles. It demonstrates the application of REIMS to a number of surgical pathologies with promising results, whilst recognizing the need for further high-quality research and acknowledging logistical limitations.

## Introduction

Many surgical specialties and procedures aim to remove abnormal or damaged tissue and leave behind healthy residual tissue. Identification of abnormal or damaged tissue is usually obtained by histopathological examination. This process takes somewhere from hours to days and results are prone to subjective interpretation^1^. If tissue identification is required intraoperatively, modalities such as squash smear cytology, frozen sections, and fluid cytology may be employed to provide a preliminary diagnosis^2^. This preliminary diagnosis can then inform further surgical decision-making intraoperatively. Although frozen sections shorten the timeframe for a preliminary diagnosis, taking 20–30 min^1^, they are prone to errors that can have an adverse impact on intraoperative management^3^. Ideally, intraoperative tissue identification methods should be rapid, standardized, and diagnostically accurate^4^. Rapid evaporative ionization mass spectrometry (REIMS) is a relatively new technology that poses as a potential solution to many of the current problems with intraoperative tissue identification^1^.

Diathermy is a common surgical tool used to cut, coagulate, and achieve haemostasis^5^. It functions by passing electrical current, in different waveforms, through tissues. The resistance of the tissues results in heat production^6^ and heat vaporizes the tissue to produce surgical smoke^7^. The evaporation of biological tissues produced by diathermy heat is rich in molecular ions, such as phospholipids, which can then be analysed with a mass spectrometer^8^. The mass spectrometric profile produced by using diathermy on tissue allows for tissue identification data comparable to histopathological analysis^1^. Coupling of the electrosurgical unit (diathermy) with REIMS technology is termed the ‘intelligent knife’ (iKnife)^9^.

REIMS technology has a growing body of evidence demonstrating successful applications in the surgical field. This systematic review aims to discuss the uses, advantages, disadvantages, and reported outcomes of REIMS technology for intraoperative tissue identification in surgical practice. In doing so, it will identify areas that would benefit from further research and improve awareness among the surgical community.

## Methods

The study protocol was designed with reference to the Preferred Reporting Items for Systematic Reviews and Meta-Analyses Protocols Statement (PRISMA-P)^10^ and the associated explanation document^11^. The protocol was registered in the PROSPERO international prospective register of systematic reviews before commencing the review (CRD42024546741)^12^. The manuscript has been developed in line with the PRISMA statement, with the PRISMA checklist included in the *Supplementary material*^13^.

### Search strategy and inclusion criteria

A robust search strategy (see the *Supplementary material*) was designed in collaboration with an experienced research librarian (Cheryl Hamill) and peer-reviewed by another (Glynis Jones). A search of MEDLINE (Ovid) 1946 to current, Emcare (Ovid) 1995 to current, Embase (Ovid) 1974 to current, and Web of Science (Clarivate) 1997 to current was performed on 7 August 2025. The start date for each database search is based on the commencement date of the database itself. The search strategy prioritized sensitivity for maximum retrieval of relevant records, using a combination of free-text (keyword) and thesaurus terms. No grey literature searching was performed and no limits were applied to the search. After deduplication of references, 344 citations were identified, to which the inclusion and exclusion criteria were applied.

#### Inclusion criteria

- Studies published in peer-reviewed journals AND
- Full texts available in any language, but with adequate English translation AND
- The study design is RCT OR non-randomized study of intervention OR observational studies (case-control study OR cohort study) OR case series with ≥5 patients AND
- The study evaluates the use of REIMS/iKnife technology in surgery/a surgical environment or application to a surgical pathology AND
- REIMS is used on humans or human tissue (both *in vivo* and *ex vivo*)

#### Exclusion criteria

- Studies that were case reports OR case series with <5 patients OR literature reviews OR conference abstracts OR
- Studies without adequate full-text English translation OR
- Studies evaluating REIMS/iKnife technology in a non-surgical environment and application to a non-surgical pathology OR
- Animal studies

### Data extraction and variables

After completion of the literature search, studies were uploaded to Covidence systematic review software^14^ for title/abstract and full-text screening. A data extraction template (see the *Supplementary material*) was designed and piloted by the authors before the extraction process. For both title/abstract and full-text screening, two authors (A.R.J.B. and A.D.) screened studies independently in duplicate, with a third author (J.L.) arbitrating the decision if disagreement occurred. The level of inter-reviewer agreement during title/abstract and full-text screening is summarized using Cohen’s κ coefficient. Data extraction was also performed in duplicate by two of A.R.J.B., A.D., and J.L., with arbitration by the third author in cases of disagreement.

### Study risk-of-bias assessment

Included studies were all assessed for risk of bias. This assessment occurred at the study level, by two independent reviewers, and in the case of disagreement was arbitrated by discussion amongst authors. The QUADAS-2 tool^15^ was used to assess risk of bias, given the included articles evaluated the diagnostic accuracy of REIMS analysis.

### Synthesis methods

Given the variety of pathologies, surgical specialties, study designs, and data interpretation models applied to REIMS, studies were not deemed homogenous enough to conduct a meta-analysis. Indeed, pooling data from diverse non-randomized studies into a meta-analysis is not recommended^16^. As such, a systematic, qualitative narrative synthesis has been performed. Information from included studies has been presented in tables, as well as within the manuscript text, to explain their characteristics and findings.

## Results

### Study screening

**Fig. 1:**
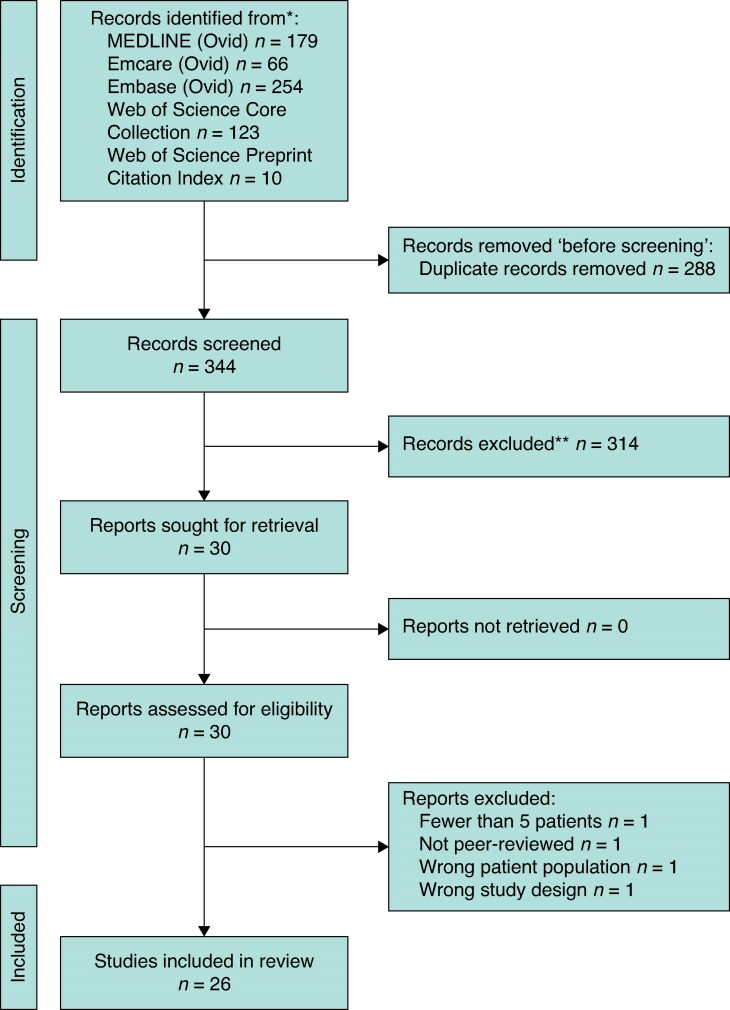
PRISMA flow diagram detailing the literature search process used to identify included studies

*Fig. 1* illustrates the process of screening using a PRISMA flow diagram^13^. The screening process was performed using Covidence software. A total of 344 records were identified after deduplication and underwent title and abstract screening, of which 30 articles progressed to full-text screening. A total of 26 studies then met the inclusion criteria and were included in this systematic review.

The level of agreement between investigators during title/abstract and full-text screening (assessed using Cohen’s κ coefficient) is shown in *Table 1*. This process demonstrated almost perfect agreement^17^ in both title/abstract and full-text screening.

**Table 1:** Agreement between reviewers during screening

Screening reviewer A	Screening reviewer B	Cohen’s κ coefficient	
		Title and abstract screening	Full-text screening
A.R.J.B.	A.D.	0.94604	1.0

### Study characteristics and results of individual studies

A total of 26 studies met the inclusion criteria and were included in the review. Studies originated from seven countries (Canada^18–25^, the Netherlands^26–29^, Belgium^30^, the UK^31–39^, Hungary^9^, China^40^, and Australia^41^). One study did not specify the country of origin^42^. Included studies were published between 2013 and 2025. REIMS was applied to eight surgical specialties, of which the most common was general surgery (9 studies), followed by plastic surgery (7 studies). Twenty-three of the included studies discussed REIMS in the context of cancer, with the most frequently studied pathology being basal cell carcinoma (BCC), which was the subject of six papers.

Of the 26 included studies, 14 used both *ex vivo* and *in vivo* data, whilst the remaining 12 used *ex vivo* data only. Twenty-two studies prospectively collected data, whilst one was both retrospective and prospective, and three studies were retrospective.

As a ‘gold standard’ comparator for REIMS analysis, 23 studies relied on histopathological analysis to determine the performance of REIMS. Davies *et al*.^32^ used previously reported biomechanical and biochemical variables for comparison. Yau *et al*.^41^ compared REIMS analysis of burn tissue with the intraoperative judgement of a burns surgeon. Hendriks *et al*.^29^ used confirmed overall survival data for included patients.

Among included studies, a total of 1825 patients contributed to the analysis. Two included studies^40,41^ did not specify the number of patients, but instead reported the number of samples, which contributed an additional 78 tissue samples. One included study^25^ reported patient numbers for the *ex vivo* component of the study (91 patients), but reported sample numbers for the *in vivo* data (43 resections). Of the 23 studies reporting complete patient numbers, 1302 patients were analysed *ex vivo*, 54 were analysed *in vivo*, and for 484 patients it was not specified if their analysis occurred *ex vivo* or *in vivo*. A number of studies analysed a large number of *ex vivo* patients and then applied this data set to analyse a smaller number of *in vivo* patients as a proof of concept.

Of the 26 included studies, two articles^39,41^ made their data available via a public repository and a further five articles^24,27–29^  ^,31^ had data attached in the Supplementary material of the paper. Eight papers^9,26,32,34–36,38,40^ made data available on request by contacting the corresponding author. There were ten articles without any data availability statement, which also did not include any data in the Supplementary material. One article stated data from the study were not available.

Study characteristics and findings are summarized in *Table 2*.

**Table 2:** Summary of findings

Reference and year	Surgical specialty	Disease process	Study design	Data interpretation model	Data set size	Model validation	Reported qualitative outcomes	Reported quantitative outcomes
Janssen *et al*.^18^2020	Plastic surgery	BCC	*Ex vivo* and *in vivo*	PCA-LDA	596 spectra	Five-fold cross-validation	Intraoperative (*in vivo*) REIMS showed significantly lower accuracy than *ex vivo* REIMSOf the three included *in vivo* cases, sterility whilst using REIMS was never breached	*Ex vivo* REIMS classification showed 92% accuracy, 86% sensitivity, 97% specificity, with 95% PPV*In vivo* REIMS showed 50% accuracy
Santilli *et al*.^19^2021	Plastic surgeryGeneral surgery	BCCBreast cancer	*Ex vivo*	SSL using ‘relative patch algorithm’	464 spectra	Four-fold cross-validation	An SSL task (learning from BCC data and applying this to breast cancer data) suggests shared features between skin and breast data	For the model trained with skin data and validated on breast cancer data, accuracy was 92.7%, sensitivity 88.5%, specificity 96.4%, AUC 92.7%
Connolly *et al*.^20^2024	Plastic surgery	BCC	*Ex vivo* and *in vivo*	- MLP- PCA-LDA- BNN- Experimental model (involving GADF, GASF, MTF)	11 100 spectra	Five-fold cross-validation	Of the non-experimental models used to analyse data, BNN was the highest performing modelTransforming iKnife signals into images and using self-supervision may outperform traditional modelsFatty acids and glycerophospholipids showed differential abundance between cancerous and benign tissue	*Ex vivo* data:- MLP model: accuracy 73.8%, sensitivity 59.4%, specificity 83.7%, AUC 78.6%- PCA-LDA model: accuracy 69.7%, sensitivity 66.0%, specificity 73.5%, AUC 73.9%- BNN model: accuracy 72.1%, sensitivity 60.5%, specificity 83.7%, AUC 81.1%- Experimental model (with conversion to images) with pretraining: accuracy 73.5%, sensitivity 63.1%, specificity 83.9%, AUC 81.6%
Santilli *et al*.^21^2020	Plastic surgery	BCC	*Ex vivo*	- PCA-LDA- Experimental model (deep learning network with modified symmetrical autoencoder architecture)	190 spectra	Four-fold cross-validation	Insufficient smoke or low-intensity signals make it more difficult to predict sample classVariations in mass spectra can occur due to depth of burn or body site of tissueIf sub-band analysis is applied, the range of 500–900 m/z yields the best accuracy differentiating normal *versus* BCC tissue. However, accuracy is improved when lower sub-bands are also included (suggesting they contain important information for classification)The ideal deep learning network model had five layers. Less layers decreased classification accuracy, more layers may overfit training data	PCA-LDA model:- Accuracy 85.1%, sensitivity 72.9%, specificity 91.0%Augmentation of PCA-LDA model data improved mean performanceDeep learning model means: accuracy 96.6%, sensitivity 100%, specificity 95%
Vaysse *et al*.^26^2024	Surgical oncology	Soft tissue sarcoma	*Ex vivo* and *in vivo*	PCA-LDA	1200 spectra	Leave-one-patient-out cross-validation	Fatty acids, phospholipids, and cardiolipin were predominant in cut mode; triacylglycerols were predominant in coagulate mode	With cut and coagulate mode data combined, sarcoma tissue was correctly classified 98.2% of the time, normal tissue correctly classified 98.6% of the time, overall classification rate 95.5%
Van Hese *et al*.^30^2022	Neurosurgery	Glioma	*Ex vivo*	PCA-LDA	124 spectra	Five-fold cross-validation	Glycophospholipids, phosphatidic acids, sphingolipids, and fatty acids were identified as contributing to class separationDifferences between classes due to different mass peak ratios, rather than the presence or absence of specific lipid speciesA monopolar model was applicable to bipolar electrocautery	Overall accuracy of 87.9% for a model including classes: normal, astrocytoma grade 2, astrocytoma grade 3, glioblastoma, oligodendroglioma grade 2, oligodendroglioma grade 3In high-grade gliomas, overall correct classification rate 97.0%, with 99.3% sensitivity and 100% specificity. For margin detection, bulk glioblastoma tumour was identified as 100% glioblastoma, normal brain as 0% glioblastoma, and the invasive region (margin) was detected as 10% glioblastoma via REIMS, which was histopathologically quantified as 10.7% glioblastoma cells
Mason *et al*.^31^2023	General surgery	Colorectal cancer	*Ex vivo*	- PCA-LDA- OPLS-DAUnivariate method:- ANOVA	1013 spectra	Leave-one-patient-out cross-validation	No single lipid metabolite is capable of acting as a biomarker of disease and comparisons require multivariate modelling to assess differences in relative abundanceRelative abundance of lipids by subclass increases at initiation of adenoma formation (except phosphatidylcholines, which decrease). Abundance of phosphatidylglycerols increases throughout carcinogenesisBased on the mucosal lipidome, REIMS was able to predict the presence of nodal metastasis with the same NPV as preoperative cross-sectional imaging (78.0% with REIMS *versus* 78.8% with imaging)	REIMS identified colorectal carcinoma with accuracy of 91.1%, sensitivity 86.4%, specificity 95.4%, PPV 90.7%, NPV 93.1%One source of misclassifications were adenomas predicted to be carcinomas. Clinically both adenomas and carcinomas require excision, so if considered as one group accuracy increases to 93.5%, sensitivity 91.2%, specificity 96.7%, PPV 97.5%, NPV 88.6%
Balog *et al*.^9^2013	Surgery in general	Various subtypes of cancer	*Ex vivo* and *in vivo*	PCA-LDAUnivariate method:- Kruskal–Wallis test	2933 spectra	Leave-one-patient-out cross-validation	It is the profile of lipid species rather than any specific biomarker that accounts for histological specificityREIMS was able to identify the origin of metastatic tumours both *in vivo* and *ex vivo*Multivariate models comparing *in vivo* and *ex vivo* spectra showed no separation, suggesting that *in vivo* modelling is robust and representativeMetastatic deposits demonstrate clear metabolic separation from surrounding healthy tissue, in contrast to primary tumours where a transition zone is detected	*In vivo*, REIMS confirmed the result of postoperative histopathology (when differentiating cancer *versus* healthy tissue) with 96.2% accuracy, sensitivity 97.7%, specificity 96.5%. 3.5% false positive and 2.3% false negative rate
Ma *et al*.^40^2021	Neurosurgery	Glioblastoma multiforme	*Ex vivo*	- PCA- PLS-DA	Not stated	Not stated	A difference in lipid composition between tumour and control samples was identified. Most lipids showed higher intensities in tumour group, which is consistent with increased lipogenesis in tumour cells	REIMS showed overall accuracy of 94.7%, sensitivity of 95.4%, specificity 93.3%, and AUC 0.968
Fooladgar *et al*.^22^2022	Plastic surgery	BCC	*Ex vivo*	- PCA-LDA- BNN model- A deep model (similar to BNN, but without Bayesian output and uncertainty estimation)	693 spectra	Not stated	A model with higher sensitivity that detects all cancer at margins is preferredPCA-LDA is not improved by uncertainty filtering, but applying uncertainty filtering to BNN models improves model accuracy	PCA-LDA:Accuracy 69.7%, sensitivity 66.0%, specificity 73.5%, AUC 73.9%BNN model with uncertainty filtering: 75.2% balanced accuracy, 74.1% sensitivity, 77.3% specificity, 82.1% AUCExcluding highly uncertain samples from decision-making can improve decisions and overall sensitivity (88.2% sensitivity if 20% of highly uncertain spectra rejected)
Davies *et al*.^32^2021	Vascular surgery	Aortic aneurysm	*Ex vivo*	PLS-DA	Not stated	Two-fold cross-validation	Patient sex and source of normal tissue (cadaveric *versus* elective surgical patient) did not discriminate in the model	REIMS was able to discriminate between normal and aneurysmic aortic tissue with 88.7% mean accuracy and 85.1% mean precision using a second generation model
Vaysse *et al*.^27^2020	General surgery	Breast cancer	*Ex vivo* and *in vivo*	PCA-LDA	689 spectra	Leave-one-patient-out cross-validation	The main discriminators of class were triglycerides for adipose tissue and fatty acids, palmitic acid, and oleic acid for stroma*Ex vivo* REIMS models enabled recognition of adipose tissue and stroma *in vivo*	*Ex vivo* data:Stroma *versus* adipose *versus* tumour was classified with 92.6% accuracy
Mason *et al*.^33^2020	General surgery	Rectal cancer	*Ex vivo* and *in vivo*	- PCA- OPLS-DAUnivariate method:- ANOVA	266 spectra	Leave-one-patient-out cross-validation	PCA demonstrated no clustering based on sex, age, or resection type (radical *versus* endoscopic *versus* local excision)No patient safety concerns were identified when using REIMS coupled with TAMISREIMS was able to distinguish layers of bowel wall during TAMIS dissection, using the distinctive lipid composition at each layer	*Ex vivo*:Overall accuracy of 86.8%, sensitivity 94.2%, specificity 91.7% when differentiating normal *versus* adenoma *versus* cancerNormal *versus* disease (tumour or adenoma): accuracy 91.4%, sensitivity 89.7%, specificity 93.4%, PPV 94.2%, NPV 88.3%*In vivo*:REIMS identified involved margins with 90% accuracy, NPV for tumour 95%
Yau *et al*.^41^2022	Plastic surgery	Burns	*Ex vivo*	- PCA- OPLS-DA	109 spectra	Not stated	Excised burn tissue showed significantly lower concentrations of free fatty acids, monoacylglyceride, lysophosphatidylglycerol, and lysophosphatidylethanolamineConcentrations of lactosylceramide and cholesterol esters were significantly higher in excised burn tissue compared with control tissueThere is a difference in lipid profiles between excised burn skin and control skin	REIMS showed distinct separation between burn tissue and control tissue, with AUC 1.0, sensitivity 100%, and specificity 100%
Phelps *et al*.^34^2018	Gynaecology	Ovarian cancer	*Ex vivo* and *in vivo*	PCA-LDAUnivariate methods:- Wilcoxon’s rank-sum- Kruskal–Wallis test	3387 spectra	Leave-one-patient-out cross-validation	The three most significant MS peaks contributing to separation of ovarian cancer from normal samples were various forms of phosphatidic acid and phosphatidylethanolamine. These peaks were higher in ovarian cancer tissue	Ovarian cancer *versus* normal tissue: ovarian cancer was correctly classified in 97.6% of samples, with 97.4% sensitivity and 100% specificityOvarian cancer *versus* borderline ovarian tumour *versus* benign tumour: overall correct classification 83.1% (that is at differentiating degree of invasion)No *in vivo* data
Davies *et al*.^35^2022	Cardiothoracic and vascular surgery	Type A aortic dissection	*Ex vivo*	- PCA- PLS-DAUnivariate method:- Dunn Kruskal–Wallis test	Not stated	Two-fold cross-validation	PCA analysis showed separation between true lumen wall *versus* false lumen wall *versus* dissection flap, based on biomechanical properties and glycosaminoglycan levels. The false lumen wall had lower levels of glycosaminoglycan	REIMS was able to discriminate false lumen wall tissue from other tissue types with 72.4% accuracy and 69.3% precision
St John *et al*.^36^2017	General surgery	Breast cancer	*Ex vivo* and *in vivo*	PCA-LDAUnivariate method:- Mann–Whitney *U* test	359 spectra	Leave-one-patient-out cross-validation	Normal tissue demonstrated high-intensity spectra in the phospholipid and triglyceride range using cut mode and predominantly in triglyceride range when using coagulate modeTumour tissue has increase in phospholipid and decrease in triglyceride range in both cut and coagulate modesPhospholipid species elevated in cancer (*versus* normal tissue) were glycerophospholipids	*Ex vivo* data: model accuracy 95.8%, sensitivity 90.9%, specificity 98.8%A model created using ‘cut’ mode only has slightly higher accuracy and sensitivity and specificity compared with coagulate only or a combined model (cut and coagulate)*In vivo* data: inadequate sample size to determine diagnostic accuracy, but 99.3% of intraoperative spectra were interpretable by the *ex vivo* model (good overlap between *in vivo* and *ex vivo* models)
Alexander *et al*.^37^2017	General surgery	Colorectal cancer	*Ex vivo* and *in vivo*	PCA-LDAUnivariate method:- ANOVA	104 spectra	Leave-one-patient-out cross-validation	Cancerous tissue overexpressed long-chain phosphatidylserines and bacterial phosphatidylglycerolsWhen data were modelled against age and sex, it was not possible to build a strong predictive model for these factors (that is these factors did not significantly influence REIMS). However, males and patients >70 years old did demonstrate clusteringAnatomically, rectal samples were distinctly clustered from remaining colonic specimens, suggesting a discrete mucosal lipidome	Normal mucosa *versus* cancer: 90.5% accuracy, 86.7% sensitivity, 92.4% specificityNormal mucosa *versus* adenoma: 97.5% accuracy, 85.7% sensitivity, 98.6% specificityCancer *versus* adenoma: 94.4% accuracy, 78.6% sensitivity, 97.3% specificityREIMS was also able to distinguish histological subtype, tumour staging, prognostic data (for example differentiation, lymphovascular invasion)No *in vivo* data
Marcus *et al*.^38^2022	Gynaecology	Endometrial cancer	*Ex vivo*	PCA-LDAUnivariate method:- Kruskal–Wallis test	453 spectra	Leave-one-patient-out cross-validation	Larger sample size PCA-LDA improved diagnostic accuracyEndometrial cancer had up-regulated ceramides and phospholipids (phosphatidic acid, phosphatidylethanolamines, and phosphatidylserines)	Endometrial cancer *versus* normal tissue with iKnife: 86% accuracy, 81% sensitivity, 91% specificity, PPV 90%, NPV 84%
Tzafetas *et al*.^39^2020	Gynaecology	Cervical cancer	*Ex vivo* and *in vivo*	- LASSO- LDAUnivariate method:- ANOVA	209 spectra	Leave-one-patient-out cross-validation	MS peaks contributing to separation of cancer *versus* normal tissue: sphingomyelins, phosphatidic acids, phosphatidylethanolamines, phosphatidylglycerols, phosphatidylcholine, and phosphatidylinositols	A REIMS model classifying normal *versus* HPV/CIN *versus* cervical cancer: 100% accuracy, sensitivity 100%, specificity 100%
Balog *et al*.^42^2015	General surgery	Gastrointestinal cancer	*Ex vivo* and *in vivo*	PCAUnivariate method:- Kruskal–Wallis test	186 spectra	Leave-one-patient-out cross-validation	Gastric mucosa (both healthy and adenocarcinoma) featured phospholipids in 600–900 m/z range, whilst submucosa featured triglyceride and phosphatidylinositol species in 850–1000 m/z range*In vivo* data agreed with *ex vivo* data	*Ex vivo* data (normal *versus* cancerous tissue): 95% specificity, 88.5% sensitivity
Vaysse *et al*.^28^2022	Ear, nose, and throat	Oral squamous cell carcinoma	*Ex vivo* and *in vivo*	PCA-LDA	185 spectra	Leave-one-patient-out cross-validation	Metabolic profiles for soft tissue and tumour showed distinctive profiles	REIMS was capable of detecting 10% tumour cells in a sample with 83% sensitivity and 82% specificity. By detecting a small proportion of tumour cells in a sample, REIMS may assist with tumour margin recognition in highly infiltrative malignancy*Ex vivo* REIMS analysis had cross-validation accuracy of 96.8%No *in vivo* data presented
Kaufmann *et al*.^24^2024	General surgery	Breast cancer	*Ex vivo*	PCA-LDA	260 spectra	- Leave-one-patient-out cross-validation- Leave-one-site-out cross-validation- Five-fold cross-validation	Triglycerides dominated the spectra acquired from normal tissue, but were mostly absent from tumour tissue spectra. Tumour spectra had a relatively high abundance of phospholipidsCosine similarities between cancerous tissue tested was lower than normal adipose tissue, especially in coagulate modeCertain triglycerides are distinctly elevated in coagulate modeMetabolism of linoleic acid was enhanced in triple-negative breast cancer compared with ER/PR-positive cancer	Model using a training set from two sites and testing it on a third site, with leave-one-patient-out cross-validation: 98.6% correct classification rate, 2.8% false negative, 0.7% false positiveIf a more targeted model based on 11 selected metabolites is applied, correct classification rate is 98%. This may decrease the impact of spectral variability on classification
Radcliffe *et al*.^23^2025	General surgery	Breast cancer	*Ex vivo* and *in vivo*	- iForest- OC-PCA- GODS- KGODS	2149 spectra	Four-fold cross-validation	Incorporating healthy *ex vivo* samples into the training set increased balanced accuracy during cross-validation for all modelsUnsupervised anomaly detection methods could enhance current methods for REIMS analysis	For models trained on intraoperative data only:- iForest: balanced accuracy 70.2%, sensitivity 90.9%, specificity 49.4%- OC-PCA: balanced accuracy 81.0%, sensitivity 90.9%, specificity 71.0%- GODS: balanced accuracy 77.1%, sensitivity 67.9%, specificity 86.4%- KGODS: balanced accuracy 80.6%, sensitivity 79.4%, specificity 81.8%For models trained on intraoperative and healthy *ex vivo* data:- iForest: balanced accuracy 86.1%, sensitivity 88.6%, specificity 83.6%- OC-PCA: balanced accuracy 83.6%, sensitivity 84.8%, specificity 82.4%- GODS: balanced accuracy 83.1%, sensitivity 77.3%, specificity 88.9%- KGODS: balanced accuracy 84.8%, sensitivity 93.0%, specificity 76.6%
Farahmand *et al*.^25^2025	Plastic surgery	BCC	*Ex vivo* and *in vivo*	- PCA-LDA- MLP- Foundation model for assessing cancer tissue margins (FACT)	11 100 spectra	Three-fold cross-validation	There is correlation between misclassified specimens and sample noise	PCA-LDA model: balanced accuracy 69.7%, sensitivity 66.0%, specificity 73.5%MLP model: balanced accuracy 73.9%, sensitivity 67.8%, specificity 79.9%FACT model: balanced accuracy 77.5%, sensitivity 72.2%, specificity 82.8%
Hendriks *et al*.^29^2025	Neurosurgery	Glioblastoma multiforme	*Ex vivo*	PCA-LDA	87 spectra	Five-fold cross-validation	Metabolite patterns differed between patients with short-term survival *versus* prolonged survivalThere is potential for REIMS to enhance survival predictions and contribute to personalized treatment strategies	PCA-LDA model correctly classified 97.7% of samples into short term (0–12 months) *versus* prolonged (>12 months) survivalWhen measuring an unidentified sample, short term survival was distinguished with 66.7% correct classification and prolonged survival with 69.4% correct classification

BCC, basal cell carcinoma; PCA-LDA, principal component analysis-linear discriminate analysis; REIMS, rapid evaporative ionization mass spectrometry; PPV, positive predictive value; SSL, self-supervised learning; AUC, area under the curve; MLP, multilayer perceptron; BNN, Bayesian neural network; GADF, Gramian angular difference field; GASF, Gramian angular summation field; MTF, Markov transition field; iKnife, intelligent knife; OPLS-DA, orthogonal partial least squares discriminant analysis; NPV, negative predictive value; PCA, principal component analysis; PLS-DA, partial least squares discriminant analysis; TAMIS, transanal minimally invasive surgery; MS, mass spectrometry; LASSO, least absolute shrinkage and selection operator; LDA, linear discriminate analysis; HPV/CIN, human papilloma virus/cervical intraepithelial neoplasia; iForest, Isolation Forest; OC-PCA, one-class principal component analysis; GODS, generalized one-class discriminative subspaces; KGODS, kernelized generalized one-class discriminative subspaces.

### Risk of bias in included studies

The 26 included studies were assessed for risk of bias using the QUADAS-2 tool^15^ by two independent reviewers and in the case of disagreement was arbitrated by discussion amongst authors. QUADAS-2 was chosen because the vast majority of included studies commented on the diagnostic accuracy of REIMS in identifying tissue types. Among artificial intelligence (AI)-based diagnostic accuracy studies, QUADAS-2 is the most commonly used risk-of-bias assessment tool^43^. It is acknowledged that there are significant limitations with this tool when applied to AI diagnostic studies. AI diagnostic accuracy studies can cause issues within all four domains of QUADAS-2^43^, but, specifically in the case of REIMS, reporting on the ‘flow and timing’ category may unfairly determine a high risk of bias. A common methodology amongst REIMS analyses is to perform cross-validation, where a proportion of available data is intentionally left out from the model-training process and left available for model testing/validation at a later stage. As such, there is scope for this to be interpreted as all patients not being included in the analysis, which would contribute to a high risk of bias in the ‘flow and timing’ category. Consequently, in this study, a concerted effort was made to not penalize studies for this, so when studies excluded patients from model training, but later included them in model testing, this was not considered to be a high risk of bias. An AI-specific extension to QUADAS-2 has been recommended^44^, but is not yet available, limiting quality assessment to existing tools.

**Fig. 2:**
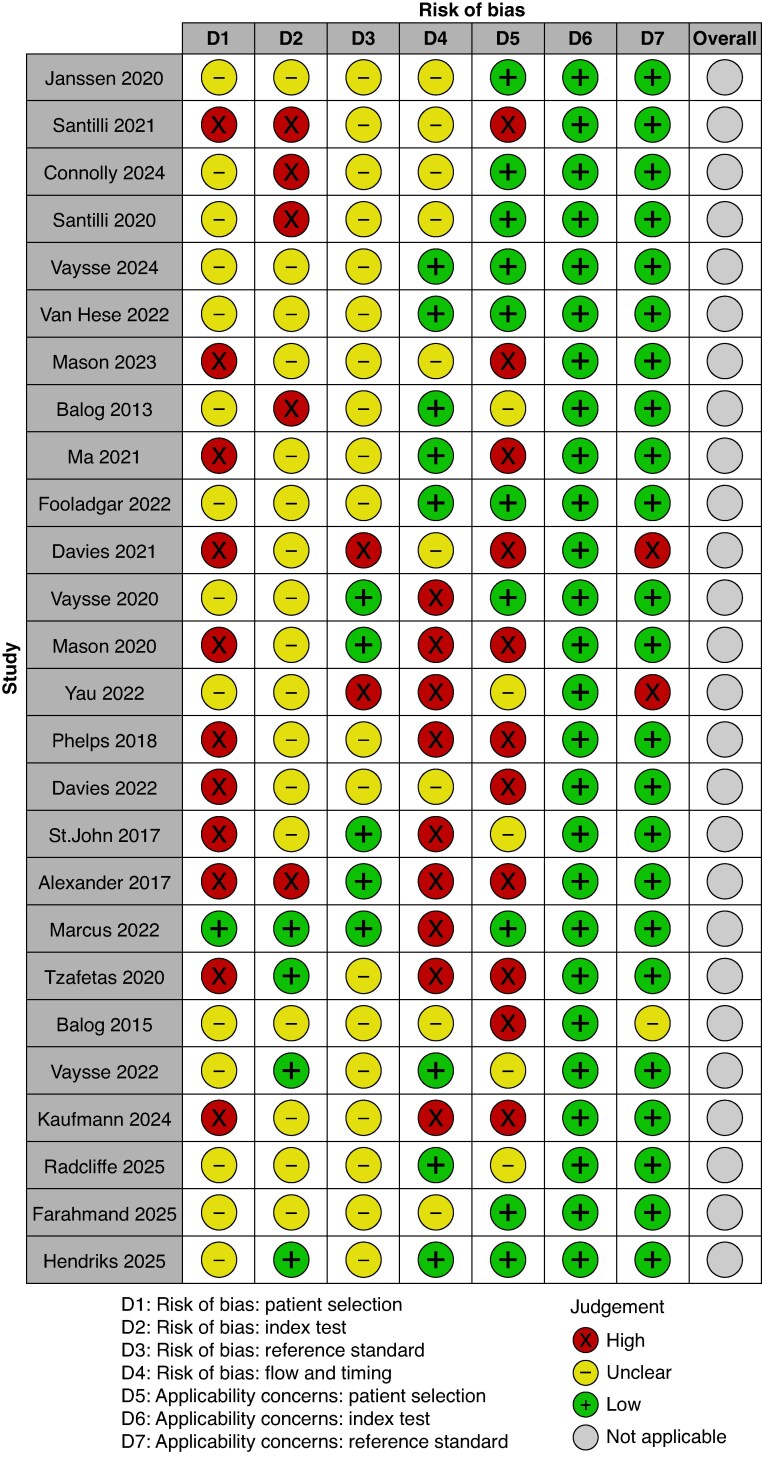
QUADAS-2 risk-of-bias assessment for included studies

The robvis tool^45^ was used to create a visual representation of study risk of bias and this is presented in *Fig. 2*. This assessment demonstrated significant risk of bias among most of the included studies, suggesting the need for further high-quality research into REIMS technology. A number of studies did not comment on how their patient cohort was selected (for example, consecutive or random patients), making the patient selection domain unclear. Many studies did not include all recruited patients in the final analysis, resulting in a high risk of bias in the ‘flow and timing’ category. This was often on account of poor spectral quality or high background noise, leading to exclusion of the affected spectra from analysis.

### Results of syntheses Reported qualitative outcomes

#### Statistical methods used for REIMS analysis

REIMS analysis generates a large amount of molecular data, which must then be classified using predictive statistical methods. This allows for allocation of the data points to a particular class, for example normal or cancerous tissue. Both univariate and multivariate statistical methods can be employed to analyse REIMS data. A univariate analysis looks at each variable in isolation and, in the context of mass spectrometry, univariate models are often applied to determine relevant ‘information-rich’ features of the data set^46^. A common application for univariate methods in REIMS is to determine metabolites responsible for class discrimination^33,34^, for example determining peaks that differ between normal and cancerous tissue^36^. Univariate analyses may work to reduce the number of features considered in a subsequent multivariate model, by focusing on those showing statistical significance in initial univariate tests^46^.

Multivariate analysis incorporates all variables simultaneously and assesses relationships among them^47^. A multivariate classification method may be supervised or unsupervised. Unsupervised methods uncover general structural patterns and relationships within a data set, helping develop hypotheses and explore data sets without predefined data labelling^48^. Supervised methods require data to be labelled and summarize variables in a way that explains their relationship with another variable^49^. In the context of REIMS, unsupervised analyses such as principal component analysis (PCA) are often initially applied for data reduction, to obtain an overview of the variance of metabolic profiles^28^. PCA reduces the number of observed variables into a smaller number of principal components, which account for the majority of variance in the data set^37^. This helps interpret a large complex metabolomic data set^50^. Subsequent supervised analyses such as linear discriminate analysis (LDA) are then used to maximize the separation between different classes, allowing classification of the data^37,51^. Implementing both univariate and multivariate analysis methods in mass spectrometry is recommended to optimize the relevant data extracted from a data set^46^.

Ten studies reported using univariate statistical methods for data analysis. These were primarily implemented to identify differential peaks between normal and pathological tissue. The most commonly implemented methods were the Kruskal–Wallis test and ANOVA. ANOVA is used to compare the means of three of more independent data sets that meet the parametric assumptions, whilst the Kruskal–Wallis test is the equivalent test for data sets that do not meet parametric assumptions^52^. The decision to apply parametric or non-parametric methods to REIMS data should be made after considering if the data set meets the parametric assumptions or not^46^.

Among included articles, 17 different multivariate statistical methods were applied for REIMS analysis. Seventeen studies utilized a PCA-LDA model of analysis and a further five articles used PCA without LDA as a component of their model. Although PCA-LDA analysis is generally considered best practice in multivariate REIMS analysis^21^, the optimal algorithm for REIMS analysis is likely dependent on the classification problem^53^ and, as such, evaluation of multiple algorithms is recommended during model development. Many included studies trained their models for binary classification tasks (for example, healthy *versus* malignant tissue)^22^, whilst others trained a subcategory classification model (for example, different types and grades of malignancy)^30^. PCA-LDA analysis appeared to perform well in both binary and subcategory analyses, for example Mason *et al*.^33^ applied PCA-LDA to both binary (no disease *versus* disease) and subcategory (cancer *versus* adenoma *versus* normal tissue) classifiers. Despite this strong performance, a number of studies tested experimental statistical methods that outperformed PCA-LDA for their specific classification task. PCA-LDA performs well for classification of groups that tend to show large differences in the molecular profiles of the samples^53^. However, when the molecular profiles of groups are less distinct, alternative machine learning methods may perform more favourably. Another common model used for analysis is partial least squares discriminant analysis (PLS-DA). PLS-DA is a ‘supervised’ version of PCA, meaning it reduces dimensionality, but has an awareness of the class labels^54^. Due to the variety of pathologies, models tested, and settings among included studies, there is insufficient data to determine the overall best performing model. However, with further research, larger sample sizes, and a consideration for the classification task required (binary *versus* subcategory), model performance may improve as the model itself is tailored to the specific pathology.

#### Data set size and model validation

Among the included articles, data set size ranges from 87 to 11 100 spectra and multiple model validation methods are implemented. Cross-validation is an essential step when developing AI algorithms, such as those used to interpret REIMS data. Cross-validation partitions the data set into independent cohorts for training and testing, allowing model performance to be evaluated, whilst minimizing bias and overfitting^55^. The most common cross-validation method applied is leave-one-patient-out cross-validation, seen in 13 of the included articles. This method leaves out all spectra from an individual patient and rebuilds the model without them. It then tests the removed spectra against the new model to determine the diagnostic accuracy of the model. This is repeated with each individual patient sample^38^. This has the benefit of providing more data available for use in training, but requires significant computational demands to calculate. An alternative methodology employed by 11 articles is k-fold cross-validation, during which the data set is divided into ‘k’ subsets, with the majority applied during model training and a subset reserved for model testing^55^. For example, in five-fold cross-validation, the data set is divided into five ‘folds’. One fold (20% of the spectra) is randomly excluded from the training set and the model is built with the remaining 80% of spectra. The 20% is used to test the model and this process is repeated five times to include all spectra^29^. Anywhere from two to five folds have been applied among included articles. A disadvantage of this approach is that the ‘test’ data set may not be truly representative of the population^55^. Three included studies did not explicitly specify their method of cross-validation. The significant range of data set sizes, along with different cross-validation models applied to available data, may account for some of the differences in model performance, despite similar pathology and experimental methodology.

#### Metabolite patterns

Overall, 18 of the 26 included studies commented on substances contributing to class separation between normal and pathological tissue analysis using REIMS. Sixteen of these studies involved lipids, one study^35^ found glycosaminoglycans to be the main class separator between true and false lumen wall tissue in type A aortic dissection, and another^28^ commented on ‘metabolic profiles’ separating tumour and soft tissue without specifying the metabolites contributing to this. Of the 16 studies commenting on lipids, 15 of these used lipid markers to distinguish cancerous tissue from normal tissue and one study^41^ used lipid markers to distinguish burnt tissue from non-burnt tissue. The ability of lipid markers to differentiate cancerous from normal tissue aligns with the understanding that tumour cells demonstrate altered phospholipid metabolism^56,57^. Specific metabolites contributing to class separation are summarized in *Table 3*.

**Table 3:** Specific substances contributing to class separation

Reference	Year	Substances contributing to class separation
Connolly *et al*.^20^	2024	Fatty acids and glycerophospholipids
Van Hese *et al*.^30^	2022	Glycophospholipids, phosphatidic acids, sphingolipids, and fatty acids
Mason *et al*.^31^	2023	Relative abundance of lipids by subclass increases at initiation of adenoma formation (except phosphatidylcholines, which decrease). Abundance of phosphatidylglycerols increases throughout carcinogenesis
Vaysse *et al*.^27^	2020	Triglycerides for adipose tissue. Fatty acids, palmitic acid, and oleic acid for stroma
Yau *et al*.^41^	2022	Burn tissue showed higher concentrations of lactosylceramide and cholesterol esters compared with control tissue, and lower concentrations of free fatty acids, monoacylglyceride, lysophosphatidylglycerol, and lysophosphatidylethanolamine
Phelps *et al*.^34^	2018	Various forms of phosphatidic acid and phosphatidylethanolamine were higher in ovarian cancer tissue
Davies *et al*.^35^	2022	Glycosaminoglycans
St John *et al*.^36^	2017	Tumour tissue has increase in phospholipid (specifically glycerophospholipids) and decrease in triglyceride range in both cut and coagulate modes
Alexander *et al*.^37^	2017	Cancerous tissue overexpressed long-chain phosphatidylserines and bacterial phosphatidylglycerols
Marcus *et al*.^38^	2022	Endometrial cancer had up-regulated ceramides and phospholipids (phosphatidic acid, phosphatidylethanolamines, and phosphatidylserines)
Tzafetas *et al*.^39^	2020	Sphingomyelins, phosphatidic acids, phosphatidylethanolamines, phosphatidylglycerols, phosphatidylcholine, and phosphatidylinositols
Kaufmann *et al*.^24^	2024	Triglycerides dominated spectra acquired from normal tissue, but were mostly absent from tumour spectra. Tumour had high abundance of phospholipidsMetabolism of linoleic acid was enhanced in triple-negative breast cancer compared with oestrogen receptor/progesterone receptor-positive breast cancer
Hendriks *et al*.^29^	2025	Short-term survival patients: more abundant oleic acid and palmitic acidProlonged survival patients: more abundant linoleic acid and stearic acid

With respect to lipid biomarkers in cancer, Van Hese *et al*.^30^ found that differences between classes were due to different mass peak ratios, rather than the presence or absence of specific lipid species. Mason *et al*.^31^ supported this conclusion, suggesting no single lipid metabolite is capable of acting as a disease biomarker, with multivariate modelling required to assess differences in relative abundance. Balog *et al*.^9^ also found that the profiles of lipid species, rather than specific biomarkers, account for histological specificity. These conclusions are supported by existing oncological research, which demonstrates that changes in lipid metabolism in tumour cells are not common to all cancer types; indeed, there can even be lipid heterogeneity within a single tumour^58^. This heterogeneity also validates the role of REIMS and the subsequent univariate and multivariate statistical analysis process in tissue detection, which allows a vast array of lipids and their relative abundance to be considered in the analysis, rather than looking for a finite number of ‘biomarkers’.

#### Safety profile

The safety profile of REIMS is largely based on the commonplace use of diathermy technology in operating theatres worldwide. Janssen *et al*.^18^ reported no breach of the surgical sterile field during *in vivo* use of REIMS. Mason *et al*.^33^ reported no patient safety concerns when REIMS was coupled with transanal minimally invasive surgery (TAMIS).

#### Diathermy settings

Surgical diathermy units convert electrical energy into heat, which creates diathermy ‘smoke’. This smoke contains 95% steam and 5% cellular debris^59^ and, as such, can be analysed with REIMS to allow tissue identification. Surgical diathermy can either involve a monopolar or bipolar circuit. Monopolar diathermy creates current flow from the diathermy device, through the body, and back to the electrosurgical unit via a dispersive electrode. Bipolar diathermy creates current flow from an active electrode to the return electrode on the opposite blade of a pair of forceps. The diathermy effect can be altered based on machine settings, with common options being ‘cut’ (uses a pure continuous sine wave of low voltage to vapourize tissue) and ‘coagulate’ (uses an intermittent sine wave at higher voltage to reduce the rate of heat rise and coagulate tissue)^59^. Santilli *et al*.^21^ determined that insufficient smoke or low-intensity signals make prediction of sample class difficult. Using diathermy in the cut mode generates significantly more surgical smoke compared with the coagulate mode^7^, which would suggest the cut mode is more appropriate for REIMS use. Interestingly, Vaysse *et al*.^26^ identified different lipid patterns generated by the cut and coagulate modes, with fatty acids, phospholipids, and cardiolipin predominant in the cut mode, whilst triacylglycerols predominated in the coagulate mode. Kaufmann *et al*.^24^ noted that certain triglycerides were distinctly elevated in the coagulate mode, suggesting that the electrosurgical mode impacts the spectral fingerprint of the tissue being analysed. This difference was more pronounced in breast cancer tissue compared with normal adipose tissue. Van Hese *et al*.^30^ determined that a monopolar REIMS model was applicable to bipolar diathermy applications.

### Reported quantitative outcomes

All included studies made some quantitative comment on the performance of REIMS technology for tissue identification, as summarized in *Table 4*. However, despite 14 studies using both *ex vivo* and *in vivo* data, only 3^9,18,33^ explicitly reported *in vivo* quantitative data. Two studies^26,39^ did not specify if their quantitative outcomes were derived from *ex vivo* or *in vivo* data (or a combination). One study^25^ used a data set containing both *in vivo* and *ex vivo* data. Across a variety of statistical methods used to interpret data, accuracy was the most commonly reported quantitative measure, with 24 studies discussing this. *Ex vivo* accuracy ranged from 69.7% to 98.6% (median 87.4%) and *in vivo* accuracy ranged from 50% to 96.2% (median 90%). Sensitivity ranged from 59.4% to 100% (median 86.4%) in an *ex vivo* setting, with one *in vivo* sensitivity value reported at 97.7%. Specificity ranged from 73.5% to 100% (median 91.0%) in an *ex vivo* setting, with one *in vivo* specificity value reported at 96.5%.

**Table 4:** Reported quantitative outcomes

Reference	Year	Setting of data	Accuracy	Sensitivity	Specificity
Janssen *et al*.^18^	2020	*Ex vivo*	PCA-LDA model: 92%	PCA-LDA model: 86%	PCA-LDA model: 97%
		*In vivo*	PCA-LDA model: 50%		
Santilli *et al*.^19^	2021	*Ex vivo*	SSL model: 92.7%	SSL model: 88.5%	SSL model: 96.4%
Connolly *et al*.^20^	2024	*Ex vivo*	- MLP model: 73.8%- PCA-LDA model: 69.7%- BNN model: 72.1%- Experimental model: 73.5%	- MLP model: 59.4%- PCA-LDA model: 66.0%- BNN model: 60.5%- Experimental model: 63.1%	- MLP model: 83.7%- PCA-LDA model: 73.5%- BNN model: 83.7%- Experimental model: 83.9%
Santilli *et al*.^21^	2020	*Ex vivo*	- PCA-LDA model: 85.1%- Experimental model: 96.6%	- PCA-LDA model: 72.9%- Experimental model: 100%	- PCA-LDA model: 91%- Experimental model: 95%
Vaysse *et al*.^26^	2024	Not specified	PCA-LDA model: 95.5%		
Van Hese *et al*.^30^	2022	*Ex vivo*	PCA-LDA model: 87.9% for all classes, 97.0% for high-grade gliomas	PCA-LDA model: 99.3% for high-grade gliomas	PCA-LDA model: 100% for high-grade gliomas
Mason *et al*.^31^	2023	*Ex vivo*	PCA-LDA + OPLS-DA model: 91.1%	PCA-LDA + OPLS-DA model: 86.4%	PCA-LDA + OPLS-DA model: 95.4%
Balog *et al*.^9^	2013	*In vivo*	PCA-LDA model: 96.2%	PCA-LDA model: 97.7%	PCA-LDA model: 96.5%
Ma *et al*.^40^	2021	*Ex vivo*	PCA + PLS-DA model: 94.7%	PCA + PLS-DA model: 95.4%	PCA + PLS-DA model: 93.3%
Fooladgar *et al*.^22^	2022	*Ex vivo*	- PCA-LDA model: 69.7%- BNN model: 75.2%	- PCA-LDA model: 66.0%- BNN model: 74.1%	- PCA-LDA model: 73.5%- BNN model: 77.3%
Davies *et al*.^32^	2021	*Ex vivo*	PLS-DA model: 88.7%		
Vaysse *et al*.^27^	2020	*Ex vivo*	PCA-LDA model: 92.6%		
Mason *et al*.^33^	2020	*Ex vivo*	PCA + OPLS-DA model: 86.8% for all classes, 91.4% for normal *versus* disease class	PCA + OPLS-DA model: 94.2% for all classes, 89.7% for normal *versus* disease class	PCA + OPLS-DA model: 91.7% for all classes, 93.4% for normal *versus* disease class
		*In vivo*	PCA + OPLS-DA model: 90% accuracy identifying involved margins		
Yau *et al*.^41^	2022	*Ex vivo*		PCA + OPLS-DA model: 100%	PCA + OPLS-DA model: 100%
Phelps *et al*.^34^	2018	*Ex vivo*	PCA-LDA model: 97.6%	PCA-LDA model: 97.4%	PCA-LDA model: 100%
Davies *et al*.^35^	2022	*Ex vivo*	PCA + PLS-DA model: 72.4%		
St John *et al*.^36^	2017	*Ex vivo*	PCA-LDA model: 95.8%	PCA-LDA model: 90.9%	PCA-LDA model: 98.8%
Alexander *et al*.^37^	2017	*Ex vivo*	PCA-LDA model: 90.5% for normal mucosa *versus* cancer, 97.5% for normal mucosa *versus* adenoma, 94.4% for adenoma *versus* cancer	PCA-LDA model: 86.7% for normal mucosa *versus* cancer, 85.7% for normal mucosa *versus* adenoma, 78.6% for adenoma *versus* cancer	PCA-LDA model: 92.4% for normal mucosa *versus* cancer, 98.6% for normal mucosa *versus* adenoma, 97.3% for adenoma *versus* cancer
Marcus *et al*.^38^	2022	*Ex vivo*	PCA-LDA model: 86%	PCA-LDA model: 81%	PCA-LDA model: 91%
Tzafetas *et al*.^39^	2020	Not specified	LASSO + LDA model: 100%	LASSO + LDA model: 100%	LASSO + LDA model: 100%
Balog *et al*.^42^	2015	*Ex vivo*		PCA model: 88.5%	PCA model: 95%
Vaysse *et al*.^28^	2022	*Ex vivo*	PCA-LDA model: 96.8%	PCA-LDA model: 83% for detecting 10% tumour cells in a sample	PCA-LDA model: 82% for detecting 10% tumour cells in a sample
Kaufmann *et al*.^24^	2024	*Ex vivo*	PCA-LDA model: 98.6% with leave-one-patient-out cross-validation		
Radcliffe *et al*.^23^	2025	*Ex vivo*	Models trained on intraoperative and healthy *ex vivo* data:iForest model: 86.1%OC-PCA model: 83.6%GODS model: 83.1%KGODS model: 84.8%	Models trained on intraoperative and healthy *ex vivo* data:iForest model: 88.6%OC-PCA model: 84.8%GODS model: 77.3%KGODS model: 93.0%	Models trained on intraoperative and healthy *ex vivo* data:iForest model: 83.6%OC-PCA model: 82.4%GODS model: 88.9%KGODS model: 76.6%
Farahmand *et al*.^25^	2025	*Ex vivo* and *in vivo*	PCA-LDA model: 69.7%MLP model: 73.9%FACT model: 77.5%	PCA-LDA model: 66.0%MLP model: 67.8%FACT model: 72.2%	PCA-LDA model: 73.5%MLP model: 79.9%FACT model: 82.8%
Hendriks *et al*.^29^	2025	*Ex vivo*	PCA-LDA model: 97.7%		

PCA-LDA, principal component analysis-linear discriminate analysis; SSL, self-supervised learning; MLP, multilayer perceptron; BNN, Bayesian neural network; OPLS-DA, orthogonal partial least squares discriminant analysis; PLS-DA, partial least squares discriminant analysis; LASSO, least absolute shrinkage and selection operator; LDA, linear discriminate analysis; PCA, principal component analysis; iForest, Isolation Forest; OC-PCA, one-class principal component analysis; GODS, generalized one-class discriminative subspaces; KGODS, kernelized generalized one-class discriminative subspaces.

## Discussion

To the best of the authors’ knowledge, this is the first systematic review investigating the role of REIMS and iKnife technology in surgery. This systematic review captured 26 studies exploring the role of REIMS technology in a surgical environment. A robust assortment of both qualitative and quantitative outcomes was reported and a systematic narrative synthesis of data was performed.

Intraoperative tissue identification using REIMS has the potential to provide a significant step forwards for the surgical field. Incomplete excision of cancerous lesions increases both patient morbidity and healthcare costs^60^. For BCC, the most common pathology involved among included studies, a widely accepted target for incomplete excision rates is <5%^61^. In reality, this figure varies widely^62–64^, with many centers reporting higher rates of incomplete excision. Incomplete BCC excision margins are associated with 31–41% recurrence, compared with 1% recurrence where margins are clear^60^. The rates of incomplete excision and recurrence will vary in comparison with other types of tumour. Intraoperative sampling of margins has the potential to decrease the rate of incomplete tumour excision. Emergency burns surgery aims to debride burnt skin until healthy tissue is reached, at which point skin grafts or dressings can be applied^65^. Of the available debridement methods, tangential excision is the ‘gold standard’, which sequentially shaves off burn eschar until a viable wound bed is exposed^66^. This process is heavily reliant on surgeon experience. In tangential excision, both excessive and inadequate debridement have significant consequences for the patient. Conservation of the deep dermis limits the area to be subsequently grafted, results in less scar tissue formation, and improves the texture of the graft^67^. Conservation of underlying subcutaneous fat also results in better cosmetic outcomes^68^. However, adequate removal of non-viable tissue leaves a wound bed that is less prone to infection and reduces the generation of systemic inflammatory mediators^68^. Even with advanced diagnostic methods to guide the assessment of burn depth (such as infrared photography, ultrasonography, and laser Doppler imaging), determining the ideal extent of debridement remains a difficult task^69^. The potential to guide burns debridement, by providing an intraoperative assessment of tissue viability, is another promising application of REIMS.

In addition to the applications in cancer, burns, and aortic surgery described in the included articles, there are numerous other surgical disciplines that have the potential to benefit from REIMS. Orthopaedic and spinal surgery has identified a role for intraoperative tissue classification, to guide surgical navigation and alleviate malpositioning of hardware^70^, but REIMS has not yet been explored in this field. There has also been research into the coupling of REIMS technology with robotic surgery platforms, which can further increase the accuracy of dissection^71^. REIMS can also rapidly classify bacterial species with high accuracy^72^, suggesting potential benefits during surgical debridement of infection, where identification of causative pathogens may facilitate earlier treatment with appropriate antimicrobial agents.

With 23 of the included 26 studies focusing on REIMS in the context of cancer detection, there are implications of this pathology on the quantitative outcomes required of any new diagnostic tool. Sensitivity relates to a test correctly diagnosing a patient with disease as positive, whilst specificity is the ability of a test to correctly diagnose a patient without disease as negative^73^. A sensitive test is important in cancer, to ensure that, if residual disease is present, the surgeon is alerted to this so further tissue can be resected. This may reduce the re-excision rate due to incomplete margins, which have implications such as delaying adjuvant treatment, poor cosmetic outcomes, and increased risk of local and distant disease recurrence^74^. The cost of false negatives is also much higher than false positives when applied to cancer margin detection^22^, favouring a highly sensitive test. The quantitative outcomes of REIMS have largely been determined compared with ‘gold standard’ histopathology in the included studies and it is important to remember that REIMS is providing intraoperative information on tissue type to guide decision-making, but is not replacing the role of postoperative histopathology for definitive diagnosis.

Another promising feature of REIMS analysis in the context of malignancy is the potential to identify features that may assist with disease prognostication. Early research into REIMS suggested it could provide information on the grade and possible necrosis of tumour tissue^8^. Mason *et al*.^31^ found that REIMS was able to predict the presence of histological features associated with prognosis in colorectal cancer. REIMS predicted nodal micrometastasis with the same negative predictive value as preoperative cross-sectional imaging (78.0% with REIMS *versus* 78.8% with imaging). However, the accuracy of distinction of tumour stage (52.6%), lymphovascular invasion (57.6%), and tumour subtype (49.7%) was relatively low in this study. Balog *et al*.^9^ found that metastatic deposits showed clear metabolic separation from surrounding tissue, in contrast to primary tumours with a transition zone, meaning REIMS may help determine if a tumour is of primary or metastatic origin. Vaysse *et al*.^28^ found that REIMS was capable of detecting 10% tumour cells in a sample with 83% sensitivity and 82% specificity, which points to a role for REIMS in margin recognition in highly infiltrative malignancies. Kaufmann *et al*.^24^ identified that fatty acid metabolism was altered in triple-negative breast cancer compared with oestrogen receptor/progesterone receptor-positive breast cancers, which has important treatment implications for patients^75^. Hendriks *et al*.^29^ found that metabolite patterns differ between patients with short-term *versus* prolonged survival in the context of glioblastoma multiforme. This has the potential to enhance survival predictions and help personalize treatment plans.

One issue identified with the included studies is the lack of *in vivo* quantitative data. Of the 14 studies including *in vivo* data, only 3^9,18,33^ of these reported separate quantitative *in vivo* information on the performance of REIMS. For these three papers combined, 89 *in vivo* patients contributed to the available data, significantly less than the number of *ex vivo* patients. This is especially relevant, as larger sample sizes are known to improve the diagnostic accuracy of PCA-LDA models^38^, suggesting that further research involving larger patient cohorts has the potential to generate even more accurate REIMS classifications. *In vivo* validation relies on *ex vivo* histopathological analysis as the ‘gold standard’ comparator, which introduces a fundamental difference in setting between the test and comparator. Furthermore, diathermy is destructive to tissue and subsequent histopathological analyses must resort to reviewing tissue adjacent to the *in vivo* REIMS testing site^9^. This then assumes the histopathology result is a true reflection of the adjacent burnt region, when it is known that tissue samples may be heterogeneous^29^. Of the papers evaluating *in vivo* REIMS, Balog *et al*.^9^ used continuous video monitoring of the diathermy, synchronized with REIMS data, to provide additional information regarding the tissue being cut. This was supplemented with subsequent histopathological examination along the electrosurgical dissection line. Janssen *et al*.^18^ used optical tracking to assist in surgical navigation of the diathermy electrode, but relied on visual identification of tumour and healthy tissue regions. Burnt areas were not validated by histopathology, which introduces significant limitations to the analysis by Janssen *et al*.^18^. Mason *et al*.^33^ captured video sequences of the procedure, to allow correlation of the tissue being analysed with REIMS spectra. All samples were then submitted for histopathological analysis. As such, all three papers discussing *in vivo* REIMS analysis utilized some form of visual identification of tissue type in an attempt to further evaluate *in vivo* REIMS analysis. Only two of these studies supplemented this with histopathology. The *ex vivo* nature of the ‘gold standard’ histopathological comparator and small sample size do limit these *in vivo* analyses.

There are significant practical limitations of REIMS, including financial cost and large machine size. The technology in its current form requires a central location within operating theatres due to limited mobility, as well as temperature regulation mechanisms^39^. There is also a complex process of instrument set-up, including calibration, which requires technical expertise. Surgical smoke generated by the diathermy unit must be aspirated into the REIMS machine for analysis. This process is often driven by a Venturi air pump^38^, which transports smoke via polytetrafluoroethylene (PTFE) tubing to the machine^40^. Many diathermy units have built-in smoke evacuation channels, originating near the diathermy tip, which help facilitate this process. Another limitation of the REIMS set-up is the delay between tissue contact with diathermy and REIMS detection. The large machine size and requirement for a central location mean that a significant length of tubing may be needed between the diathermy unit and the REIMS unit, especially for *in vivo* applications. A delay of between 0.7 and 2.5 s was noted by Balog *et al*.^9^ and a 1.8 s delay was noted by St John *et al*.^36^. This delay introduces latency between tissue sampling and the REIMS signal feedback, which may cause errors with interpretation or delays in the procedure. This delay can affect the usability and practical applicability of the REIMS set-up^76^, but can be minimized by optimizing tube diameter and flow rate. These logistical and technical challenges have been identified as an obstacle to routine implementation in a clinical environment^33^, but, with further development, some of these limitations may be addressed. The destructive nature of diathermy use during REIMS testing, especially on small tissue samples, can make the precise cell composition of sampling points difficult to confirm^24^. This may also impact the postoperative formal histopathological analysis of tissue^28^. Consideration must also be given to surgical cases that do not routinely use diathermy, in which a diathermy set-up would be required specifically to facilitate REIMS analysis, incurring further costs. There has been no formal analysis to date investigating the impact of REIMS technology on operating time and operating theatre turnaround. Many of the included studies had a significant risk of bias, again suggesting the need for high-quality, randomized, prospective research further evaluating the technology.

With further research into REIMS and uptake of the platform, more *in vivo* data should be generated to validate the promising *ex vivo* results. This should also help determine the optimal univariate and multivariate data analysis methods for each pathology and patient cohort. Alexander *et al*.^37^ identified that age and sex were not strongly predictive of data, although there was some clustering in males and patients >70 years of age. Davies *et al*.^32^ also found that patient sex did not discriminate in their model. Because REIMS data appear relatively unchanged by common variables such as age and sex, development of large pathology-specific REIMS databases could facilitate robust data models to improve detection accuracy. As the technology supporting REIMS analysis improves, and data analysis models are generated based on large sample numbers, REIMS interpretation could move towards a succinct ‘on screen’ assessment of the sample (red/green, pathological/normal), rather than a full spectrum of data, to assist with intraoperative decision-making.

The potential for REIMS to guide intraoperative tissue identification has been explored in a number of surgical disciplines with significant potential benefits. This systematic review has highlighted the uses, qualitative and quantitative outcomes, and limitations of REIMS in surgery to date. Future research should focus on *in vivo* applications of REIMS, with large patient numbers and in a broader range of surgical applications, to provide a more rigorous assessment of the technology and its potential benefits.

## Supplementary Material

znaf228_Supplementary_Data

## Data Availability

Additional data have not been published in a public repository. The authors agree to make the data, analytic methods, and study materials available to other researchers. These can be obtained by contacting the corresponding author.
