# Supplementary material for: Rapid evaporative ionization mass spectrometry in surgery: a systematic review
Source: Br J Surg. 2025 Nov 12;112(11):znaf228. doi: 10.1093/bjs/znaf228 (PMC12605806; doi:10.1093/bjs/znaf228)
Supplement: znaf228_Supplementary_Data [file znaf228_supplementary_data.docx]

**Rapid Evaporative Ionisation Mass Spectrometry in Surgery: A Systematic Review**

**Authors**

Dr Angus R. J. Barber (corresponding author)

Plastic Surgery Department, Sir Charles Gairdner Hospital, North Metropolitan Health Service, Perth, Western Australia, Australia

Curtin Medical School, Curtin University, Perth, Western Australia, Australia

School of Biomedical Sciences, University of Western Australia, Perth, Western Australia, Australia

[Angusbarber1@gmail.com](mailto:Angusbarber1@gmail.com)

<https://orcid.org/0000-0001-6760-9361>

Dr Alexander Dottore

Orthopaedic Surgery Department, Royal Adelaide Hospital, Adelaide, South Australia, Australia

Faculty of Medicine and Health, University of Sydney, Sydney, New South Wales, Australia

<https://orcid.org/0009-0002-9897-5529>

Dr James Leigh

Nuffield Department of Primary Care Health Sciences, University of Oxford, Oxford, United Kingdom

<https://orcid.org/0000-0002-5031-0539>

Associate Professor Mark Fear

Burn Injury Research Unit, School of Biomedical Sciences, University of Western Australia, Perth, Western Australia, Australia

<https://orcid.org/0000-0003-3163-4666>

Professor Fiona Wood

State Adult Burn Unit, Fiona Stanley Hospital, Murdoch, Western Australia, Australia

Fiona Wood Foundation, Perth, Western Australia, Australia

Burn Injury Research Unit, School of Biomedical Sciences, University of Western Australia, Perth, Western Australia, Australia

<https://orcid.org/0000-0001-5427-6588>

**Corresponding Author**

Dr Angus R. J. Barber

Email address: [Angusbarber1@gmail.com](mailto:Angusbarber1@gmail.com)

Postal address: Plastic Surgery Department, Sir Charles Gairdner Hospital, North Metropolitan Health Service, Hospital Avenue, Nedlands, Western Australia, 6009, Australia

**Supplementary Materials - Index**

| **Supplementary Appendixes** |  |
| --- | --- |
| Appendix A: PRISMA Checklist | *Page 3* |
| Appendix B: Search Strategy | *Page 4* |
| Appendix C: Data Extraction Table | *Page 7* |
|  |  |

**APPENDIX A: PRISMA CHECKLIST**

**
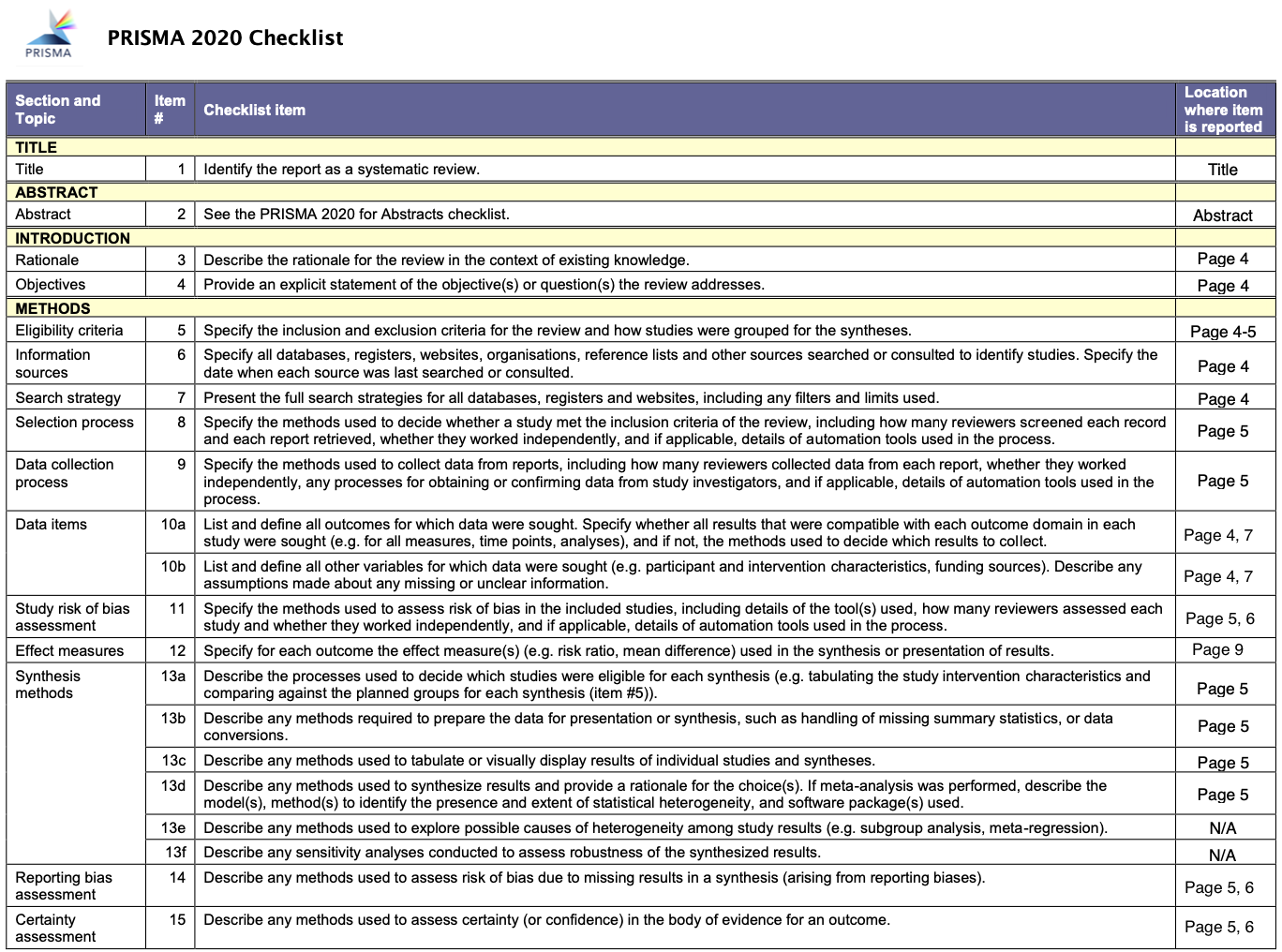
**

**
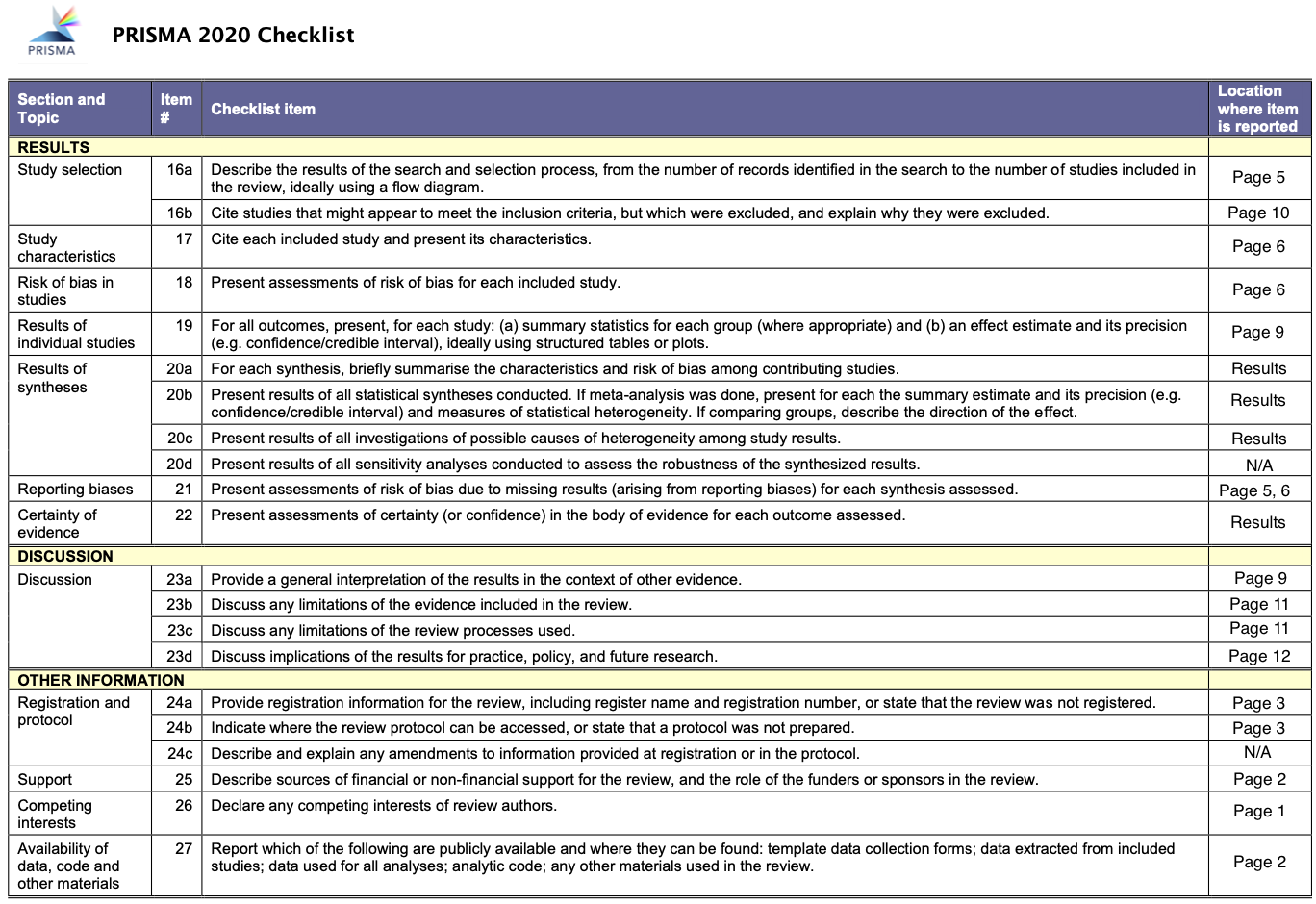
**

**APPENDIX B: Search Strategy**

**Documentation of literature searching provided in support of a systematic review**

This document describes the search methodology and results for the following systematic review:

***Rapid Evaporative Ionisation Mass Spectrometry (REIMS) in Surgery: A Systematic Review***

**Search strategy**

An experienced health librarian developed and executed search strategies using a combination of free-text (keyword) and thesaurus terms. The search was constructed around the concepts of iknife or rapid evaporative ionisation, and surgery. No search limits were applied. The initial search was developed in Medline (on the OVID platform), prioritising sensitivity for maximise retrieval of relevant records, and then transposed to Embase and Emcare (also on the OVID platform), and Web of Science. Conference papers and abstracts were excluded from the Embase and Web of Science results.

All database searches were executed on 13^th^ September 2024, AWST. The searches were rerun on the 7^th^ August 2025, AWST to identify newer publications.

**Summary of databases searched:**

| **Database and platform** | **Database coverage** |
| --- | --- |
| Medline ALL (Ovid) | 1946 to August 5^th^ 2025 |
| Emcare (Ovid) | 1995 to 2025 Week 31 |
| Embase (Ovid) | 1974 to August 5 2025 |
| Web of Science Core Collection (Clarivate)   - Science Citation Index Expanded - Social Sciences Citation Index - Arts & Humanities Citation Index - Emerging Sources Citation Index | 1997 to present *Data updated 2025-08-05* |
| Web of Science Preprint Citation Index | 1991 – present *Data updated 2025-08-01* |

**De-duplication**

The librarian performed an initial deduplication of references using EndNote 21 following the methodology documented by McGill Library^[[1]](#footnote-1)^.

**Ovid MEDLINE(R) ALL <1946 to August 7, 2025>**

| # | Query | Results from 07 Aug 2025 |
| --- | --- | --- |
| 1 | (iknife or (intelligent adj scalpel) or (intelligent adj knife) or onkoknife or surgical intelligent knife or (diathermic adj knife) or (diathermy adj knife)).mp. [mp=title, book title, abstract, original title, name of substance word, subject heading word, floating sub-heading word, keyword heading word, organism supplementary concept word, protocol supplementary concept word, rare disease supplementary concept word, unique identifier, synonyms, population supplementary concept word, anatomy supplementary concept word] | 111 |
| 2 | (rapid evaporati* ioni?ation or reims).mp. | 416 |
| 3 | exp surgical procedures, operative/ or surgery.fs. or surg*.mp. | 5,548,674 |
| 4 | (1 or 2) and 3 | 179 |

**Embase <1974 to 2025 August 7>**

| # | Query | Results from 07 Aug 2025 |
| --- | --- | --- |
| 1 | (iknife or (intelligent adj scalpel) or (intelligent adj knife) or onkoknife or surgical intelligent knife or (diathermic adj knife) or (diathermy adj knife)).mp. [mp=title, abstract, heading word, drug trade name, original title, device manufacturer, drug manufacturer, device trade name, keyword heading word, floating subheading word, candidate term word] | 207 |
| 2 | (rapid evaporati* ioni?ation or reims).mp. | 762 |
| 3 | exp surgery/ or surgery.fs. or surg*.mp. | 7,923,575 |
| 4 | (1 or 2) and 3 | 368 |
| 5 | limit 4 to conference abstracts | 114 |
| 6 | 4 not 5 | 254 |

**Ovid Emcare <1995 to 2025 Week 31>**

| # | Query | Results from 07 Aug 2025 |
| --- | --- | --- |
| 1 | (iknife or (intelligent adj scalpel) or (intelligent adj knife) or onkoknife or surgical intelligent knife or (diathermic adj knife) or (diathermy adj knife)).mp. [mp=title, abstract, heading word, drug trade name, original title, device manufacturer, drug manufacturer, floating subheading word, device trade name, keyword heading word, candidate term word] | 41 |
| 2 | (rapid evaporati* ioni?ation or reims).mp. | 133 |
| 3 | exp surgery/ or surg*.mp. | 1,747,294 |
| 4 | (1 or 2) and 3 | 66 |

**Web of Science 1997 to present *Data updated 2025-08-05***

| Search Query | Results |
| --- | --- |
| TS=(iknife or (intelligent scalpel) or (intelligent knife) or onkoknife or surgical intelligent knife or (diathermic knife) or (diathermy knife)) | 279 |
| TS=(rapid evaporative ioni?ation mass spectrometry or reims) | 999 |
| #1 OR #2 | 1226 |
| TS=(surgery) | 1488110 |
| #3 AND #4 and 23RD CONGRESS OF THE EUROPEAN SOCIETY OF CATARACT AND REFRACTIVE SURGEONS or 35TH ANNUAL MEETING OF THE AMERICAN SOCIETY OF CLINICAL ONCOLOGY or 35TH ANNUAL MEETING OF THE EUROPEAN ASSOCIATION FOR CARDIO THORACIC SURGERY EACTS or 38TH ANNUAL CTRC AACR SAN ANTONIO BREAST CANCER SYMPOSIUM or DIGESTIVE DISEASE WEEK MEETING 107TH ANNUAL MEETING OF THE AMERICAN GASTROENTEROLOGICAL ASSOCIATION or TRIOLOGIC SECTION OF THE COMBINED OTOLARYNGOLOGY SPRING MEETING (Exclude – Conference Titles) | 123 |

**Web of Science Preprints 1991 – present *Data updated 2025-08-04***

| Search Query | Results |
| --- | --- |
| TS=(iknife or intelligent scalpel or intelligent knife or onkoknife or surgical intelligent knife or diathermic knife or diathermy knife) | 4 |
| TS=(rapid evaporative ioni?ation mass spectrometry or reims) | 6 |
| #1 OR #2 | 10 |

**APPENDIX C: DATA EXTRACTION TABLE**

| Study Reference |  |
| --- | --- |
| Year of Publication |  |
| Country of Origin |  |
| Number of patients |  |
| Surgical specialty involved |  |
| Disease process of interest |  |
| Sample analysis and storage |  |
| Study method/design |  |
| Model used to interpret data (model for visualisation and/or dimension reduction) |  |
| Reported qualitative outcomes |  |
| Reported quantitative outcomes |  |
| Comparator for REIMS evaluation |  |
| Comments |  |

1. McGill Library. (n.d.) *Deduplication in EndNote*. Retrieved December 27, 2023, from <https://libraryguides.mcgill.ca/ld.php?content_id=35807581> [↑](#footnote-ref-1)
